# Selective regain of *egfr *gene copies in CD44^+^/CD24^-/low ^breast cancer cellular model MDA-MB-468

**DOI:** 10.1186/1471-2407-10-78

**Published:** 2010-03-03

**Authors:** Konstantin Agelopoulos, Burkhard Greve, Hartmut Schmidt, Heike Pospisil, Stefan Kurtz, Kai Bartkowiak, Antje Andreas, Marek Wieczorek, Eberhard Korsching, Horst Buerger, Burkhard Brandt

**Affiliations:** 1 Department of Medicine, Hematology and Oncology, University of Muenster, Muenster, Germany; 2 Institute of Pathology, University of Muenster, Muenster, Germany; 3 Department of Radiotherapy, University Hospital of Muenster, Muenster, Germany; 4 Institute of Clinical Chemistry and Laboratory Medicine, University Hospital of Muenster, Muenster, Germany; 5 Center for Bioinformatics, University of Hamburg, Hamburg, Germany; 6 Institute of Tumour Biology, University of Hamburg, Hamburg, Germany; 7 Institute of Bioinformatics, University of Muenster, Muenster, Germany; 8 Institute of Pathology, Cooperative Breast Center, Paderborn/Hoexter, Germany

## Abstract

**Background:**

Increased transcription of oncogenes like the epidermal growth factor receptor (EGFR) is frequently caused by amplification of the whole gene or at least of regulatory sequences. Aim of this study was to pinpoint mechanistic parameters occurring during *egfr *copy number gains leading to a stable EGFR overexpression and high sensitivity to extracellular signalling. A deeper understanding of those marker events might improve early diagnosis of cancer in suspect lesions, early detection of cancer progression and the prediction of *egfr *targeted therapies.

**Methods:**

The basal-like/stemness type breast cancer cell line subpopulation MDA-MB-468 CD44^high^/CD24^-/low^, carrying high *egfr *amplifications, was chosen as a model system in this study. Subclones of the heterogeneous cell line expressing low and high EGF receptor densities were isolated by cell sorting. Genomic profiling was carried out for these by means of SNP array profiling, qPCR and FISH. Cell cycle analysis was performed using the BrdU quenching technique.

**Results:**

Low and high EGFR expressing MDA-MB-468 CD44^+^/CD24^-/low ^subpopulations separated by cell sorting showed intermediate and high copy numbers of *egfr*, respectively. However, during cell culture an increase solely for *egfr *gene copy numbers in the intermediate subpopulation occurred. This shift was based on the formation of new cells which regained *egfr *gene copies. By two parametric cell cycle analysis clonal effects mediated through growth advantage of cells bearing higher *egfr *gene copy numbers could most likely be excluded for being the driving force. Subsequently, the detection of a fragile site distal to the *egfr *gene, sustaining uncapped telomere-less chromosomal ends, the ladder-like structure of the intrachromosomal *egfr *amplification and a broader range of *egfr *copy numbers support the assumption that dynamic chromosomal rearrangements, like breakage-fusion-bridge-cycles other than proliferation drive the gain of *egfr *copies.

**Conclusion:**

Progressive genome modulation in the CD44^+^/CD24^-/low ^subpopulation of the breast cancer cell line MDA-MB-468 leads to different coexisting subclones. In isolated low-copy cells asymmetric chromosomal segregation leads to new cells with regained solely *egfr *gene copies. Furthermore, *egfr *regain resulted in enhanced signal transduction of the MAP-kinase and PI3-kinase pathway. We show here for the first time a dynamic copy number regain in basal-like/stemness cell type breast cancer subpopulations which might explain genetic heterogeneity. Moreover, this process might also be involved in adaptive growth factor receptor intracellular signaling which support survival and migration during cancer development and progression.

## Background

Increased transcription of (proto-) oncogenes is frequently caused by amplification. This has already been shown for numerous genes for example in lung [1], pancreatic [2], brain [3] and breast cancer [4]. It is still under debate if this process is the dominant cancer cause and promoter of cancer progression or if distinct DNA sequence mutations have to lead the way. Clonal selection during cancer development may lead to a dominant cancer cell subpopulation with distinct chromosomal alterations. But, in most cases a heterogeneous cell population can be found within one tumour. Here, the non-ambiguous relationship between the genotype of distinct loci and the phenotype is physiological a rare event and therefore, highly selective in cancer. Although, contemporary genetics has shown that quantitative trait loci exist [5], it is unlikely that the gene dosage sensitivity for a single locus significantly changes the phenotype of normal somatic cells. However, specific gene families e.g. involved in essential signal transduction systems show such dosage sensitivity in cancer. The most prominent example for those genes are the HER receptor family, most prominent EGFR, which induce signal transduction for survival, proliferation and migration. Patients suffering from carcinoma associated with gene amplification and overexpression of EGFR tend to have more aggressive diseases. EGFR overexpression has been associated with poor prognosis in human breast cancer [6,7] and failure of endocrine therapy in breast cancer as well [8,9].

Although, overexpression of EGFR may be regulated on the transcriptional level, in many cases aberrant activation of EGFR is mediated primarily by chromosomal changes generating increased *egfr *gene copy numbers. Amplifications of the *egfr *gene can be detected *in vivo *sometimes as double minutes but in most cases formation of homologous staining regions (HSR) as ladder like amplification structures can be observed. This was seen frequently in glioblastoma multiforme where amplification and overexpression of the *egfr *gene occurs in about 40% of the cases [10].

However, cell culture models reflecting these characteristics are fairly seldom. Even more, primary tumours taken into cell culture often loose their *egfr *gene amplification and by this the originally displayed heterogeneity [11,12]. Among the remaining few cell culture models with conserved *egfr *amplifications and overexpression of the receptor the breast cancer cell line MDA-MB-468 as well as the subclone MDA-MB-468 CD44+/CD24^-/LOW ^reflect best the above described findings. For both clones, not only a intrachromosomal high copy *egfr *gene amplification can be found which is based upon one abnormal chromosome 7 but also a heterogeneous cell population which differs in the grade of this amplification. Furthermore, the grade of amplification reflects the expression of the receptor. Far beyond these genetic properties MDA-MB-468 CD44+/CD24^-/LOW ^is a valuable model for studies in breast cancer progression because it is likely to represent a basal-like/stemness cell phenotype of breast cancer. Besides the expression of cytokeratin 5/6 the subline investigated here consisted of CD44^+^/CD24^-/low ^cells which supports the hypothesis that it has the intrinsic capability for asymmetric cell division [13,14]. We therefore choose this cell line model to study the underlying mechanism as well as the kinetics of isolated *egfr *gene copy number variation.

## Methods

### Cell culture

MDA-MB-468 CD44+/CD24 -/LOW were described elsewhere [13]. In contrary to the standard culture conditions (αMEM, 10% FBS) cells were cultured in Dulbecco's Modified Eagle Medium (DMEM) supplemented with nonessential amino acids (GIBCO/BRL), 5% fetal calf serum and antibiotics in a humidified 5% CO_2 _incubator at 37°C.

### Immunostaining

For the cell surface antigen staining, single cell suspensions were prepared by detaching 1-3 × 10^6 ^cells with 0.5 mM EDTA in phosphate buffered saline (PBS) for 5 min and washed twice with PBS at room temperature. Blocking was performed with 1% BSA in PBS for 30 min followed by incubation for 1 h on ice with the FITC labeled primary mouse monoclonal antibody EGFR 528 (Santa Cruz Biotechnology) targeting an extracellular epitope of EGFR. Two additional washing steps were followed and cells were resuspended in PBS.

### Flow cytometry

#### Fluorescence Activated Cell Sorting (FACS)

Cell fluorescence signals were determined immediately after staining using a FACSVantage SE flow cytometer (Becton Dickinson) equipped with an argon laser emission of 488 nm. FITC was identified by using a 530 band pass filter. The analysis was performed using CELLQUEST software (Becton Dickinson). A primary gate based on physical parameters (forward and side light scatter, FSC and SSC, respectively) was set to exclude debris and cell aggregates. The background level was estimated by omitting the primary antibody. For isolation of subpopulations two additional gates were set discriminating clearly a high- from a low-level EGFR-expressing population. Either isolated cells were directly used for FISH, quantitative real-time PCR and SNP arrays respectively, or after adherence and cell culture as described above.

#### Particle Analyzing System (PAS)

For flow cytometric analysis a PAS III flow cytometer (Partec, Muenster, Germany) equipped with a 488-nm argon ion laser was used. Data acquisition and evaluation were done with FlowMax software (Quantum Analysis, Münster, Germany).

### Genomic profiling

#### High resolution SNP analysis

DNA was extracted from MDA 468 high and low copy number cells and each was typed twice using GenChip^® ^Human Mapping 500 k arrays (Affymetrix^®^). The intensity values were calculated as means of the both corresponding arrays. The data set was normalized by the dChip software which uses the invariant set normalization and a hidden Markov model [15]. The SNP intensity values were calculated by referencing the means of each pair of repeated measurement to the means of a second pair repeated measurements (pairs: EGFR low vs. EGFR high). Regions of elevated or reduced copy number were calculated by DNAcopy package 1.10.0 and GLAD to identify and visualize change-points [16].

#### Quantitative real-time PCR (5' nuclease assay)

Quantitative real-time PCR for the *egfr *gene was carried out as previously described [17]. PCR reactions (40 cycles; 15 seconds denaturation at 95°C; 1 minute primer annealing and extension at 60°C) were performed for *egfr *as well as for SOD2 at least in triplicates. DNA concentrations were normalized to the single copy gene SOD2. Amplification of the *egfr *gene was measured in MDA-MB-468 CD44+/CD24 -/LOW populations directly after isolation according to their EGFR-expression by FACS as well as in populations cultured for up to 92 days after sorting.

#### Fluorescence *in situ *hybridization (FISH) on metaphase and interphase nuclei

Dividing cells were blocked in metaphase by addition of colcimide for at least 4 h before harvest. Interphase nuclei preparation was performed directly from these cultures. Standard cytogenetic techniques were used for harvesting and slide preparation.

FISH was carried out as previously described [17]. In brief, probes were denatured for 5 min at 70°C in 70% formamid-0.6×SSC. Hybridization to the metaphase as well as interphase spreads was carried out overnight at 37°C in a 50% formamid-1×SSC-10% dextran sulfate solution in the presence of Cot-1-DNA (Gibco) and HPL-DNA (Sigma). Post-hybridization washes were performed at 45°C in 50% formamide-2×SSC. The chromosomes/nuclei were counterstained with DAPI, and images were captured with an Olympus BX61 microscope connected with a digital camera system DP50 (Hamburg, Germany).

### Cell cycle/Proliferation analysis

Progression of cell cycle phases was determined using the BrdU quenching technique according to the protocol of Böhmer and Ellwart [18]. Briefly, prior the above described sorting procedure cells were incubated for 18 h in 10 ml culture medium in the presence of 24 μg BrdU and 100 μg desoxycytidin. After cell sorting the cellular DNA of the subpopulations was stained for 1 h with 1.2 μg Hoechst 33342 and 1.5 μg ethidium bromide per milliliter Tris/NaCl buffer containing 0.1% Nonidet P40 and 0.2% bovine serum albumine (BSA).

For flow cytometric analysis a PAS III flow cytometer (Partec, Muenster, Germany) equipped with a mercury arc lamp for UV excitation was used. Ethidium bromide excitation was measured at 620 nm and Hoechst 33342 fluorescence at 455 nm. Data acquisition and evaluation were done with FlowMax software (Quantum Analysis, Muenster, Germany).

### SDS-PAGE and western blot analysis

Protein separation was done using 10% polyacrylamide separation gels. Samples were diluted in SDS-sample buffer, heat denatured at 95°C for 5 min and loaded onto the gel under the following running conditions: 84 V for 1 h and 130 V until the dye front reached the edge of the gel. The molecular size standard was the peqGOLD protein-marker V (Peqlab, Erlangen, Germany). For Western Blot analysis separated samples (20 μg of protein) were transferred to a PDVF membrane under semi-dry conditions. Alpha-Tubulin, Akt, phospho-Akt (Ser473), EGFR, Erk, phospho-Erk (Thr202/Tyr204), CD44, CD24 and cytokeratin 5 were detected using the following primary antibodies: rabbit anti-human alpha-tubulin polyclonal Ig (Cell Signaling, Danvers, MA, USA), rabbit anti-human Akt polyclonal Ig (Cell Signaling), rabbit anti-human phospho-Akt polyclonal Ig (Cell Signaling), rabbit anti-human EGFR polyclonal Ig (Santa Cruz Biotechnology, Santa Cruz, USA), rabbit anti-human p44/42 MAP-Kinase polyclonal Ig (Cell Signaling), rabbit anti-human phospho-p44/42 MAP-Kinase polyclonal Ig (Cell Signaling), rabbit anti-human CD44 monoclonal Ig (abcam, Cambridge, United Kingdom) and mouse anti-human CD24 monoclonal Ig (dianova, Hamburg, Germany). Bands were visualized using the enhanced chemiluminescence detection system and X-ray films (both GE Healthcare, Uppsala, Sweden) in accordance to the manufacturer's instructions. X-ray films were digitized (GS-700 imaging densitometer, Bio-Rad) and processed (Quantity one, Bio-Rad). For quantitative analysis of the Erk and phospho Erk signal intensities, the signals of Erk1 and Erk2 were combined. For each Western Blot the signal intensities were normalized to the MDA-MB-468 CD44+/CD24 -/LOW high cultured in DMEM supplemented with 5% FCS (lane 1). Each reaction was performed in biological triplicates.

## Results

**Figure 1:**
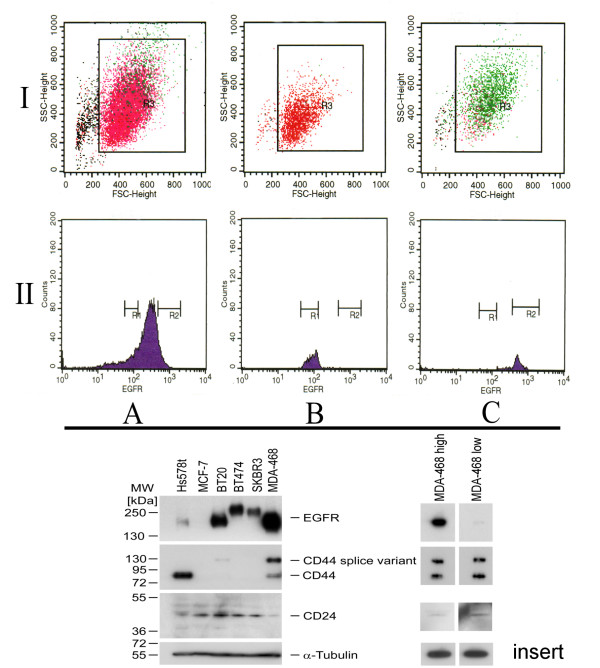
**Isolation and re-analysis of low (B) and high (C) EGFR expressing subpopulations of the breast cancer cell line MDA-MB-468 CD44+/CD24 -/LOW (A)**. Isolation was done using a FACSVantage SE flow cytometer (Becton Dickinson). A primary gate (I; R3) based on physical parameters (forward and side light scatter, FSC and SSC, respectively) was set to exclude debris and cell aggregates. Two additional gates (II; R1, R2) were set discriminating clearly a high- from a low-level EGFR-expressing population. The analysis was performed using CELLQUEST software (Becton Dickinson). Insert: Western blotting showed that used MDA-MB-468 clone was positive for CD44 and negative for CD24 which is in agreement with the stemness cell line Hs578t. The same findings could be shown for the two isolated MDA-MB-468 subclones with low high egfr copy numbers. Other used breast cancer cell lines like MCF-7, BT 20, BT 474 and SKBR 3 did not show this phenotype irrespective of their EGFR expression.

**Figure 2:**
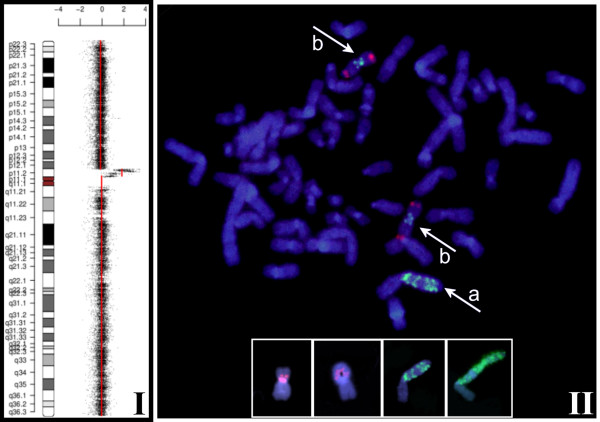
**Aberrations of chromosome 7 in MDA-MB-468CD44+/CD24 -/LOW**. I) High resolution analysis by GeneChip Human Mapping 500 K arrays revealed a lot of aberrations occurring in MDA-MB-468 CD44+/CD24 -/LOW low as well high EGFR expressing cells. Based on DNAcopy and GLAD statistical package analysis statistical significance for a difference in gene copy could only be estimated for the indicated region containing the *egfr *gene on chromosome 7p. 2 II). *Egfr *FISH (green fluorescence) displays the *egfr *gene amplification based upon one chromosome with multiple *egfr *gene copies (white arrow; a) in MDA-MB-468 CD44+/CD24 -/LOW metaphase spreads. The amplicon is equal-spaced in a ladder like structure and the concerning telomere 7ptel (red fluorescence) is lost. Isochromosomes 7p are frequent findings (white arrows; b).

**Figure 3:**
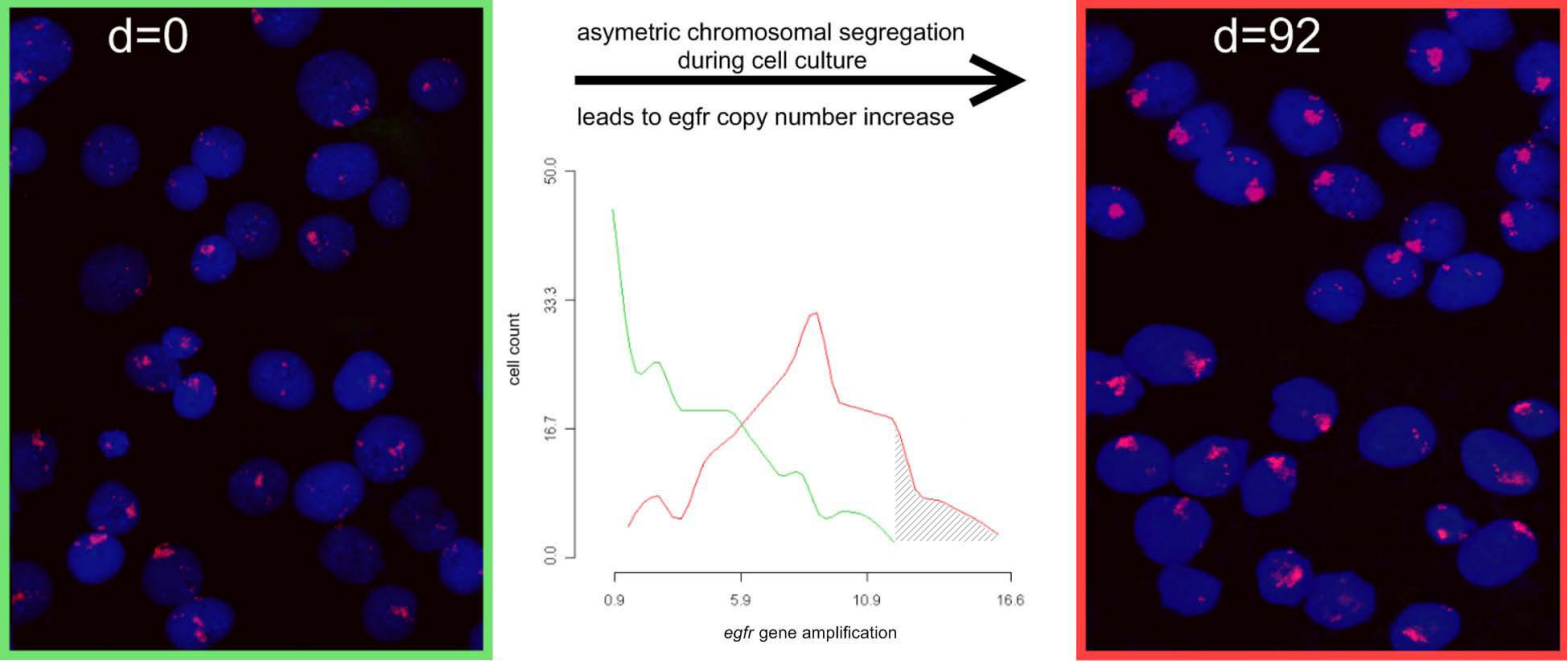
**Increase of gene copy number of the *egfr *gene in the breast cancer cell line MDA-MB-468 CD44+/CD24 -/LOW during cell culture**. FISH analysis on interphase nuclei was done using an *egfr *specific probe counterstained with DAPI. The *egfr *gene amplification of a low EGFR-expressing population (isolated by cell sorting) is increasing from the day of sorting (d = 0) to day 92 of culture. Quantification of in situ hybridization was done by measuring fluorescence areas as single spots were not available in all cases. Here we give an example displaying clearly this increase, as significant higher fluorescence intensities were obtained for cultured cells (T-Test: N1 and N2 = 200; P < 0.0001). Histograms showing the distribution of amplification within the heterogeneous cell populations (green = d0; red = d92) were derived using R and the akima package. Upward diagonals indicate new cells which were not present directly after sorting. Results were confirmed using qPCR.

**Figure 4:**
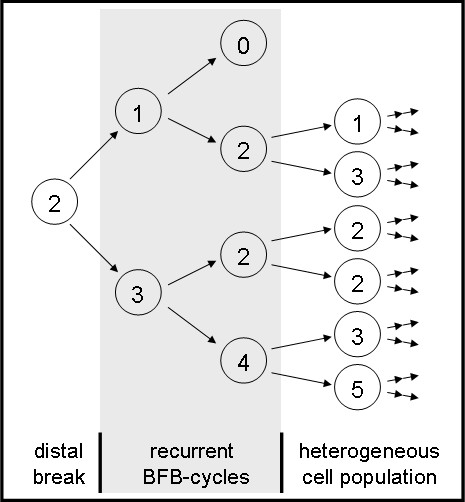
**Scheme illustrating gene amplification and heterogeinity driven by asymmetric chromosomal segregation during BFB-cycles**. After an initial break uncapped chromosomes may fuse and form a bridge during anaphase. Breakage of this bridge will form two different daughter cells one with amplification and one with deletion of the marker gene (digits indicate gene copy numbers). As chromosomes remain uncapped recurrent cycles will promote amplification as well as heterogeneity of the cell population. By this cells with decreased, normal and increased gene copy numbers persist simultaneously within the cell population.

Using cell sorting by flow cytometry a low and a high EGFR expressing MDA-MB-468 CD44^+^/CD24^-/low ^subpopulation was separated and confirmed by reanalysis (Fig 1, insert Western Blott for CD44 and CD24). In both populations standard CGH and SNP array analysis revealed a gain of chromosome 7p11-14, the chromosomal localization of *egfr*. All SNP in the chromosomal segment between 54,498,075 and 57,357,367 which spans the gene locus of *egfr *showed an increased copy number. But, the SNP within this homogeneous segment in term of copy number showed a significant different gene copy number between high and low expressing cells. No other statistically significant difference in the SNP ratio profiles could be found as determined by DNAcopy and GLAD package statistical analysis (Fig 2). This result was confirmed by the quantitative real time PCR (qPCR) assays. The high level expressing population was characterized by an average copy number of 85 (median 85; range 50-145) whereas 27 *egfr *copies (median 27; range 10-83) could be shown in the low level expressing cells. However, these gene copy numbers could only be obtained immediately after sorting due to the fact that during cell culture the copy numbers increased (Fig 3). Therefore no stable population showing an intermediate *egfr *copy number could be cultured long term for continuative analyses. Additionally, comparison of gene copy numbers obtained by FISH at different time points of cell culture revealed that the MDA-MB-468 CD44^+^/CD24^-/low ^populations remain heterogeneous at the *egfr *locus (Fig. 3). The *egfr *specific FISH signals in a population ranged from diploid spots through multiple but still countable signals up to huge clouds per cell. Despite this heterogeneity a signal shift towards higher copy numbers could be seen in respect to the culture period. During 92 days of cell culture FISH analysis revealed a relative *egfr *copy number shift within the cell population from 7 to 16. This was confirmed by qPCR where sorted cells started with 10 gene copies and ended up with 19 copies of the *egfr *gene. This shift as well as the observed heterogeneity may be based on asymmetric chromosomal segregation within breakage fusion bridge cycles which brings about cells with increased and decreased *egfr *gene copy numbers (Fig 4).

**Figure 5:**
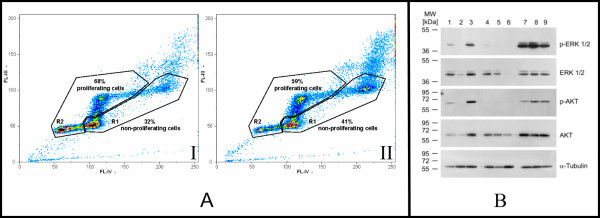
**Functional analysis of MDA sublines**. (A)Two parametric BrdU/Hoe33258 (Fl-IV) versus EtBr (Fl-III) fluorescence density plots of MDA-MB-468 CD44+/CD24 -/LOW cells displayed clearly a higher proliferation rate of low (I) EGFR expressing cells (n = 3; mean R1/R2 = 32%/68%) when compared with high (II) EGFR expressing cells (n = 3; mean R1/R2 = 41%/59%). (B) Western blot analysis of erk 1/2 (p44/p42 MAP - Kinase), phospho-erk (Thr202/Tyr204), Akt and phospho-Akt (Ser473) in MDA-MB-468 CD44+/CD24 -/LOW high (lanes 1 - 3), MDA-MB-468 CD44+/CD24 -/LOW low (lanes 4 - 6), and SK-BR-3 (lanes 7 - 9). Lanes 1, 4, and 7 represent protein expression of cells cultured in DMEM medium supplemented with 5% FCS; proteins in lanes 2, 5, and 8 are from cells cultured under 0.1% FCS for 24 h; and proteins in lanes 3, 6, and 9 are from cells stimulated with EGF (100 nM) for 30 minutes after FCS starvation for 24 h. For quantitative evaluation of western blot analysis see, Additional file 1.

Cell cycle analysis revealed that cells with intermediate *egfr *copy numbers went through mitosis slightly more rapid than those with high copy numbers (Fig 5a). Therefore, clonal effects mediated through growth advantage of cells bearing higher *egfr *gene copy numbers being the driving force in the development of a higher over all *egfr *gene amplification might be less likely. In addition, in metaphase spreads of MDA-MB-468 CD44^+^/CD24^-/LOW ^we found always no telomere on abnormal chromosomes 7p harbouring increased *egfr *copy numbers. The *egfr *gene amplification was organized equal spaced in a ladder like structure and additionally no double minutes have been detected (Fig 2). Therefore it could be hypothezised that the increase of gene copy numbers might be a consequence of asymmetric chromosomal segregation during recurrent breakage- fusion- bridge-cycles. The heterogeneity in *egfr *gene copy numbers in the MDA-MB-468 CD44^+^/CD24^-/LOW ^EGFR expressors which regained high amplification supports this assumption (Fig. 3).

One of the main downstream effectors of the epidermal growth factor receptor (EGFR) is ras GTPase, an integral molecule of the mitogen activated protein kinase (MAPK) pathway which ends in the phosphorylation of extracellular signal regulated kinase (ERK). Activated ERKs translocate to the nucleus, where they phosphorylate and regulate various transcription factors leading to changes in gene expression. In particular, ERK-mediated transcription can result in the upregulation of EGFR ligands, such as EGF and TGFa, thus creating an autocrine feedback loop that is critical for Ras-mediated transformation and Raf-mediated gene expression changes. Western blot analysis of the subclones harbouring high and low *egfr *copy numbers revealed an increased sensitivity of the high *egfr *gene copy number subclone in response to EGF in as far the phosphorylation of erk 1/2 is obviously higher (Fig. 5b lane 3; for quantitative evaluation of western blot analysis see, Additional file 1) than in the low copy number subclone (Fig. 5b lane 6). Similar findings could be shown for the activation of Akt, another important downstream effector of the EGFR pathway. Taken together, the high *egfr *copy number MDA-MB-468 CD44^+^/CD24^-/LOW ^cells represent cancer cells with a higher potential for survival, esp. at metastatic sites with low growth factor concentrations as the host tissue. A phenotype that can also be shown for cell lines with an HER2 amplification and constitutive activation of HER2, e.g. SKBR3 (Fig. 5A_lane 7-9) which displays high motogenicity.

## Discussion

The cell line MDA-MB-468 contains a subclone with a stemness phenotype CD44^high^/CD24^-/low ^expression (Shown in Fig. 1, insert) [13,14]. Using standard culture conditions this stable subclone does not show any phenotypically differences to the known MDA-MB-468. Other properties like spheroidogenesis and tumorogenesis have not been tested so far. MDA-MB-468 CD44^high^/CD24^-/low ^was sorted according to their EGFR-expression in low and high expressing populations by means of flow cytometry. Subsequent analyses (FISH, qPCR) confirmed a positive correlation between EGFR expression and *egfr *gene copy numbers indicating a direct effect on gene expression which is in agreement with data from the literature for several cancer types basal like breast cancers included [1-3,19-21].

A dynamic regain of *egfr *gene copies was observed for the low copy number subclone with time of cell culture. After a period of 12 - 14 weeks the cells regained completely the high *egfr *copy numbers. It might be assumed that this regain is caused by unequal segregation of chromosomes 7p as the cell cycle velocity of low and high copy number cells where determined nearly equal (Fig. 4a) even with a slight growth advantage of the low copy number cells. A possible mechanism of this process may be found in breakage-fusion-bridge (BFB)-cycles which can drive both, amplification [22,23] as well as heterogeneity [24]. BFB-cycles are initiated by chromosomal breakage of fragile sites distal to a selected gene [25] and based upon asymmetric segregation at mitosis. In consequence, the daughter cells will show up with different gene copy numbers, one with an increased copy number and the other with a diminished one. We were previously able to show such a fragile site breaking distal from the *egfr *locus [17] which might be the initiation of BFB-cycles. After that, chromosome-ends remain uncapped in the cells (Fig. 2) what gives some evidence for the assumption that recurrent BFB-cycles further promote amplifications and increase heterogeneity. Additionally the *egfr *amplifications are equal spaced in a ladder-like-structure (Fig. 2) and no double minutes containing the *egfr *gene were detectable. Similar findings could be shown in the human epidermoid cancer cell line A431 (own results, data not shown). Both cell lines show an intrachromosomal egfr amplification with the above mentioned findings but only A431 shows an additional polysomy of chr7. Egfr gene amplification reaches in both cell lines up to approx. 150 copies per cell. Due to the polysomy in A431 the gene copies per each chromosome do not reach the level which can be found in MDA-MB-468. These copy numbers are much higher than the ones which are seen in vivo and might thus be due to cell culture conditions. Nevertheless the structure of the egfr amplification in both cell lines is pointing towards BFB cycles as the more likely underlying mechanism for both the *egfr *amplification and the observed heterogeneity in *vitro *[23], and conceivably also in *vivo*.

In vivo, intratumoral genetic heterogeneity is not just only a well-known characteristic of numerous cancers but often confounds a precise diagnosis and leads to therapy resistance of the cancer. In this context the efficiency of antibodies targeting EGFR and small-molecule inhibitors impairing EGFR tyrosine kinase activity have to be discussed [26].

Nevertheless, we and other groups reported on the existence of a field cancerization in the human breast showing that the *egfr *aberrations were detected throughout an individual lobule or duct within histologically normal mammary epithelium with or without adjacent carcinoma [17,27-30]. Such a model comprising stem/progenitor cells and field cancerization could account for phenotypic heterogeneity within individual mammary tumors, since tumors would be composed of tumor stem/progenitor cells as well as other more differentiated progeny generated through aberrant differentiation. In this context a supporting mechanism for generating heterogeneity within clonal populations might be specifically based on *egfr *amplifications in stem/progenitor cells due to chromosomal breakage at a fragile site at chromosome 7p15. It might be concluded from the data mentioned here that EGFR is tightly involved in normal and cancer stem/progenitor cell survival, conceivably as self-renewal. Therefore, aberrations of the *egfr *gene might be events that predispose cancer cells or even its precursors for longevity and therapy resistance.

The importance of the regain of *egfr *copy numbers is also shown by our data on erk and akt phosphorylation. The main downstream effectors of EGFR are the ras GTPase, initiating the phosphorylation of extracellular signal regulated kinase (ERK) and PI3-kinase/akt. Therefore, the expression level of EGFR constitutes the sensitivity of the cancer cells to extracellular signalling. This is rather diverse in the metastatic cascade a cancer cell has to traverse during progression of the disease and the settlement in low oxygen pressure tissues as bone marrow and the brain. The resulting signals regulate Ras-mediated transformation, Raf-mediated gene expression changes, and akt/mTOR phosphorylating activity which are crucial for growth, survival, and migration of cancer cells. Therefore, the regain of *egfr *copies might strongly contribute to the survival and outgrowth of disseminated cancer cells at the metastatic site [31].

## Conclusions

A broad range of the *egfr *gene copy numbers leading to different EGFR expression levels can be found simultaneously under normal cell culture conditions in the breast cancer cell line MDA-MB-468 CD44+/CD24-/LOW. Interestingly, by flow cytometry sorted lower copy number clones showed regain of high copy numbers of *egfr*. This regain led to a heterogeneous population with synchronous high-, intermediate- and near-diploid copy number cells. The underlying mechanism might include in part breakage fusion bridge (BFB) cycles which can drive both, increase in gene copy numbers as well as genetic heterogeneity. We show here for the first time a dynamic copy number regain which might explain genetic heterogeneity and also might be involved in regulation processes in cancer cells which support survival and migration during cancer progression. Understanding of these mechanisms *in vitro *could deepen the understanding of processes *in vivo *and improve by this both diagnosis and therapy of the corresponding cancers.

## Competing interests

The authors declare that they have no competing interests.

## Authors' contributions

KA and BB designed the study and drafted the manuscript. KA also carried out the molecular genetic studies. BG carried out the cell cycle analyses and HS carried out qPCR analyses. HP and SK performed GLAD and DNAcopy analyses.

KB, AA and MW carried out SDS page and western blot analyses. EK performed statistical analyses. HB participated in the design of the study and critically revised the manuscript.

All authors read and approved the final manuscript.

## Pre-publication history

The pre-publication history for this paper can be accessed here:

http://www.biomedcentral.com/1471-2407/10/78/prepub

## Supplementary Material

Additional file 1 **Quantitative evaluation of western blot analysis**. This file contains the quantitative evaluation of western blot analysis shown in Fig 5b. Click here for file
