# Supplementary material for: Selective regain of egfr gene copies in CD44+/CD24-/low breast cancer cellular model MDA-MB-468
Source: BMC Cancer. 2010 Mar 3;10:78. doi: 10.1186/1471-2407-10-78 (PMC2841141; doi:10.1186/1471-2407-10-78)
Supplement: Additional file 1 — Quantitative evaluation of western blot analysis. This file contains the quantitative evaluation of western blot analysis shown in Fig 5b. [file 1471-2407-10-78-S1.PDF]

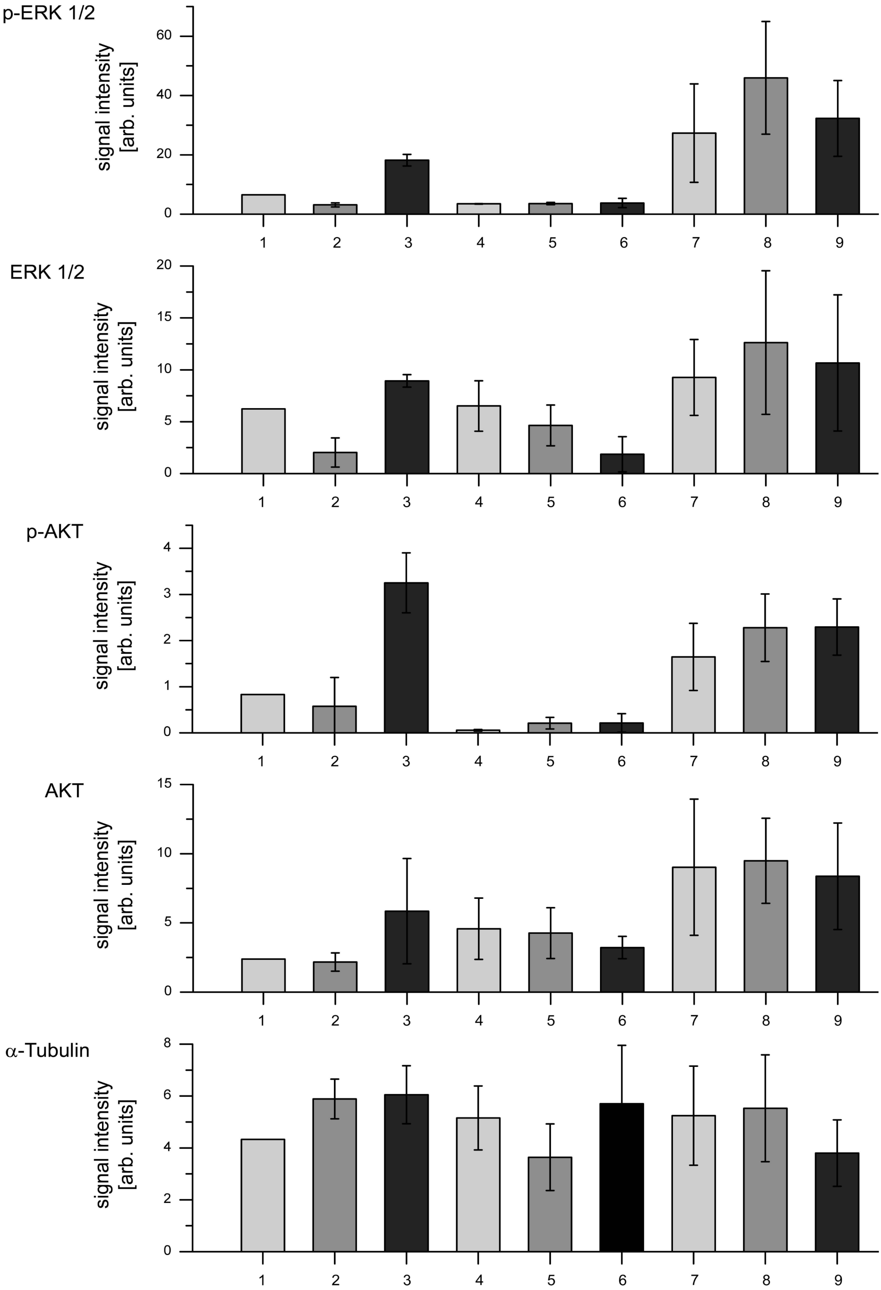

Additional material file:
Quantitative Western Blot analysis for the denoted proteins in MDA-MB-468 CD44+/CD24 -/LOW high (1 – 3), MDA-MB-468 CD44+/CD24 -/LOW low (4 – 6), and SK-BR-3 (7 - 9). Columns 1, 4, and 7 represent protein expression of cells cultured in DMEM medium supplemented with 5% FCS. Columns 2, 5, and 8 represent protein expression of cells cultured under 0.1% FCS for 24h in DMEM. Columns 3, 6, and 9 represent protein expression of cells stimulated with EGF (100nM) for 30 minutes after FCS starvation for 24h. For each Western blot the signal intensities have been normalized to the signal intensity in column 1. The average protein signal intensities of three biological experiments are shown. Standard deviation is indicated by vertical error bars. For comparison the signal intensities, quantitative analysis of alpha-Tubulin expression levels are shown.
